# Epigastric Distress Caused by Esophageal Candidiasis in 2 Patients Who Received Sorafenib Plus Radiotherapy for Hepatocellular Carcinoma: Case Report

**DOI:** 10.1097/MD.0000000000003133

**Published:** 2016-03-18

**Authors:** Kuo-Hsin Chen, Meng-Tzu Weng, Yueh-Hung Chou, Yueh-Feng Lu, Chen-Hsi Hsieh

**Affiliations:** From the Department of Surgery (K-HC), Department of Internal Medicine (M-TW), Department of Anatomical Pathology (Y-HC), Division of Radiation Oncology, Department of Radiology, Far Eastern Memorial Hospital, New Taipei City (Y-FL, C-HH), Department of Electrical Engineering (K-HC), Department of Chemical Engineering & Materials Science, Yuan-Ze University, Taoyuan (M-TW), Department of Medicine (C-HH), and Institute of Traditional Medicine, School of Medicine, National Yang-Ming University, Taipei, Taiwan (C-HH).

## Abstract

Sorafenib followed by fractionated radiotherapy (RT) has been shown to decrease the phagocytic and candidacidal activities of antifungal agents due to radiosensitization. Moreover, sorafenib has been shown to suppress the immune system, thereby increasing the risk for candida colonization and infection. In this study, we present the 2 hepatocellular carcinoma (HCC) patients suffered from epigastric distress caused by esophageal candidiasis who received sorafenib plus RT.

Two patients who had received sorafenib and RT for HCC with bone metastasis presented with hiccups, gastric ulcer, epigastric distress, anorexia, heart burn, and fatigue. Empiric antiemetic agents, antacids, and pain killers were ineffective at relieving symptoms. Panendoscopy revealed diffuse white lesions in the esophagus. Candida esophagitis was suspected. Results of periodic acid-Schiff staining were diagnostic of candidiasis. Oral fluconazole (150 mg) twice daily and proton-pump inhibitors were prescribed. At 2-weak follow-up, esophagitis had resolved and both patients were free of gastrointestinal symptoms.

Physicians should be aware that sorafenib combined with RT may induce an immunosuppressive state in patients with HCC, thereby increasing their risk of developing esophagitis due to candida species.

## INTRODUCTION

*Candida* species are part of the normal gastrointestinal (GI) flora in humans; however, patients with impaired immunity, those with chronic diseases such as cancer and diabetes mellitus (DM), patients with a history of recurrent antibiotic usage, and those receiving chemotherapy and/or radiotherapy (RT) are at increased risk of developing candida esophagitis.^1–4^

Sorafenib is a kinase inhibitor commonly used as treatment for advanced renal cell carcinoma and hepatocellular carcinoma (HCC). The drug inhibits intracellular raf kinases (CRAF and BRAF) as well as cell surface kinase receptors such as Fms-like tyrosine kinase receptor 3 (Flt-3), c-kit, Ret, vascular endothelial growth factor (VEGF)-2, VEGFR-3, and platelet-derived growth factor receptor beta (PDGFR-beta).^5,6^ One of the most common adverse effects of sorafenib is upper and lower GI distress which manifests as reflux or dyspepsia and epigastric pain, causing appetite loss, weight loss, and fatigue.^7,8^

Sorafenib suppresses CD 4^+^ T-cell activation and induces T-cell cycle arrest^9^ and has been demonstrated to significantly enhance the sensitivity of human HCC cell lines to irradiation.^10,11^ A growing body of evidence shows that irradiation has direct DNA damage-dependent effects, sending signals to distant normal tissues via a process known as the abscopal effect.^12,13^ In addition, fractionated irradiation has been shown to suppress interferon-gamma (IFN-γ)^14^ and decrease the percentage of dendritic cells (DCs) and macrophages in vivo.^15^ Moreover, radiation therapy can modulate the pharmacokinetics of anticancer drugs.^16^ These lines of evidence support the possibility that sorafenib and RT can synergistically induce an immunosuppressive state, thereby increasing the risk for infection due to candida species.

The symptoms of candida esophagitis mimic those of GI upset in patients taking sorafenib, which can lead to misdiagnosis and inadequate treatment. Herein, we present 2 patients with HCC who received sorafenib concurrently with RT as well as after completion of RT. Both patients developed candida esophagitis, the symptoms of which were initially misdiagnosed as symptoms characteristic of sorafenib-induced GI distress.

## CASE REPORT

### Case 1

**FIGURE 1:**
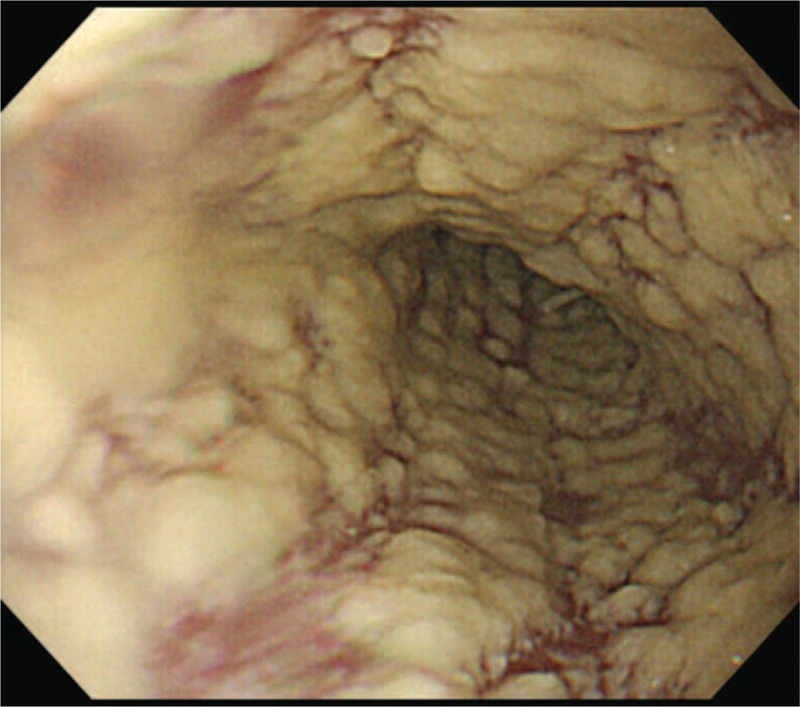
Esophagogastroduodenoscopy revealed diffuse white lesions in the esophagus characteristic of grade IV candida esophagitis according to Kodsi classification.

**FIGURE 2:**
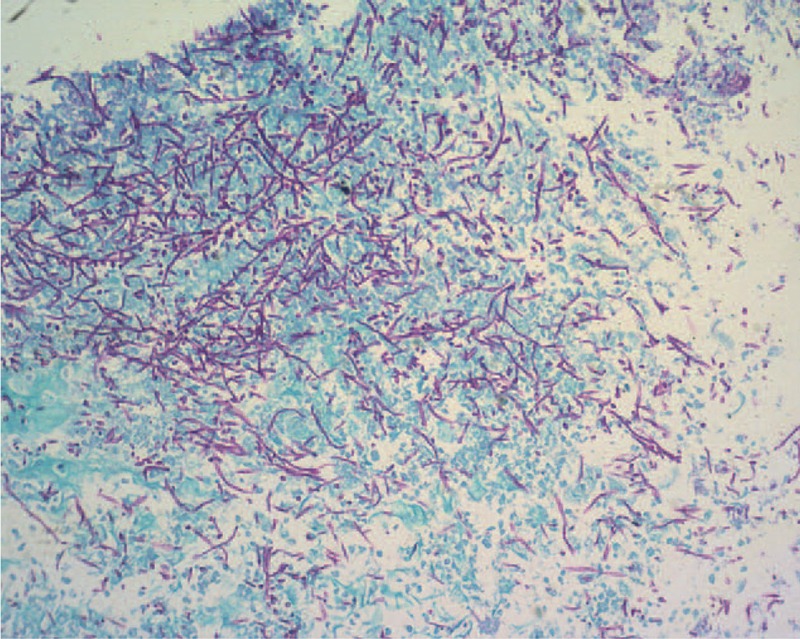
Periodic acid-Schiff (PAS) staining is indicative of candidiasis involving the squamous epithelium of the esophageal mucosa (PAS stain, magnification ×200).

**FIGURE 3:**
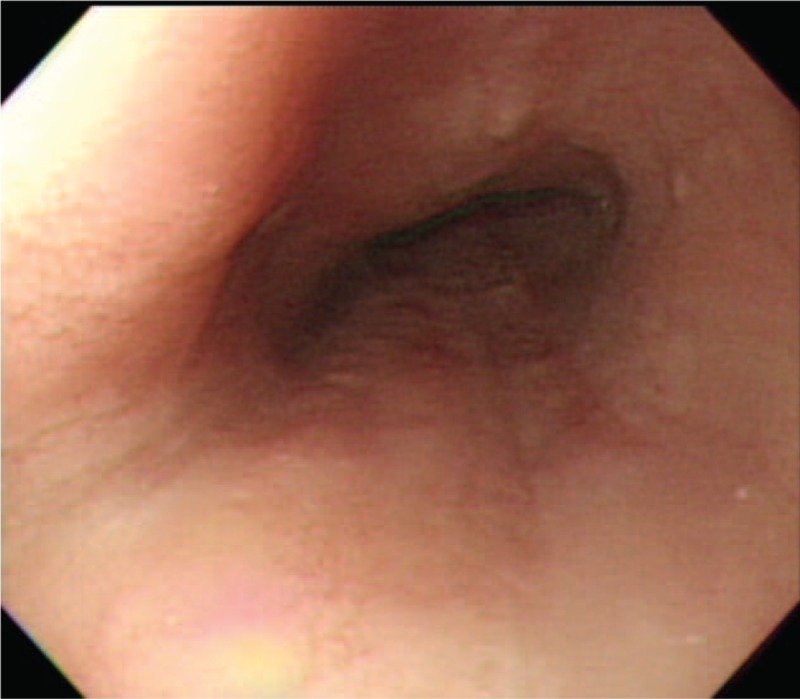
Esophagogastroduodenoscopy at 2-week follow-up shows complete resolution of candida esophagitis.

A 71-year-old man with a history of chronic hepatitis C virus infection, DM, hypertension and benign prostate hypertrophy presented with a tender mass in the right subcostal area in June 2015. Results of needle biopsy were diagnostic of metastatic HCC. Laparoscopic right hepatectomy was performed in August, 2015 and histopathologic examination of resected specimens revealed HCC. Positron emission tomography–computed tomography (PET-CT) scan showed multiple bone metastases. A total radiation dose of 45 Gy was delivered in 15 fractions to the mass located near lumbar spine (L spine) 4 to 5 and a total dose of 39 Gy was delivered in 13 fractions to the right 7th rib. The radiation course began on September 9 and was completed on October 16, 2015. Sorafenib (200 mg) 400 mg twice daily was prescribed beginning on September 7, 2015. Approximately 1 week after beginning sorafenib, the patient began to complain of hiccups, epigastric distress, anorexia, heart burn, and fatigue. Empiric antiemetic agents, antacids, and pain killers were prescribed but the symptoms persisted. Panendoscopy revealed diffuse white lesions in the esophagus (Figure 1). A diagnosis of candida esophagitis, grade IV, was made according to Kodsi classification.^17^ Periodic acid-Schiff (PAS) staining was indicative of candidiasis involving the squamous epithelium of the esophageal mucosa (Figure 2). Fluconazole (150 mg) 300 mg per os (p.o.) quaque die (qd) in 1 week was prescribed. At 2-week follow-up, panendoscopy demonstrated regression of candida esophagitis (Figure 3). Physical examination at the same follow-up visit revealed complete resolution of hiccups and epigastric distress as well as significant weight gain.

### Case 2

An 80-year-old man with goiter and benign prostatic hyperplasia underwent laparoscopic segmentectomy for segment 5 of liver in October 2014 and cholecystectomy on November 11, 2014. Alpha-fetoprotein (AFP) level decreased from 265.2 ng/ml before surgery to 3.52 ng/ml after surgery; however, at follow-up in April 2015 the AFP level was 1260 ng/ml. PET-CT scan in May 2015 revealed multiple bone metastases, including metastasis to the right scapula, the left 7th rib, the 10th thoracic (T) spine, and the 1st lumbar (L) spine but no local recurrence. Sorafenib (200 mg) 400 mg twice a day was prescribed in addition to local radiation therapy comprising a total dose of 30 Gy in 10 fractions delivered to T12 to L2 in May 2015. Grade II hand-foot syndrome was noted during the course of sorafenib and RT. In August 2015, the patient presented with persistent bone pain and an AFP level of 31,526 ng/ml. A total dose of 30 Gy in 10 fractions was delivered to T10-L1 and a total dose of 39 Gy in 13 fractions was delivered to lesions in the right scapula and left 7th rib concurrent with sorafenib (200 mg) 400 mg per day beginning in September 2015. Epigastric pain, hiccups, anorexia, and tarry stool were noted during the periods of treatment. Empiric agents were administered but the patient still complained of retrosternal pain on swallowing and persistent hiccups. Panendoscopy revealed plaques in the upper and mid esophagus indicative of candida esophagitis as well as esophageal and gastric ulcers. An 1-week regimen of fluconazole (150 mg) 300 mg p.o. qd for candidiasis and Takepron, 30 mg p.o. qd for the esophageal and gastric ulcers was administered. Physical examination at 2-week follow-up revealed complete resolution of hiccups and epigastric distress as well as significant weight gain.

The need for informed consent was waived by the Institutional Review Board of the Far Eastern Memorial Hospital (FEMH-IRB-104172-C) and retrospective data were collected after receiving approval from the Institutional Review Board of the Far Eastern Memorial Hospital (FEMH-IRB-104172-C).

## DISCUSSION

The Sorafenib HCC Assessment Randomized Protocol (SHARP) and the Asian Pacific Trial demonstrated that sorafenib (Nexavar, Bayer Pharma AG, Berlin, Germany) was associated with significantly better survival of patients with HCC than placebo.^7,8^ RT combined with sorafenib results in marked tumor shrinkage but has been shown to be associated with systemic skin reactions.^18,19^ The results of a phase II trial showed that radiation therapy plus sorafenib results in a partial response rate of 55% in patients with unresectable HCC.^20^

Grade 2 and 3 diarrhea was reported in 25% of patients who received radiation therapy concurrently with sorafenib and in 5.6% of patients who received radiation therapy after sorafenib. Moreover, grade 2/3 gastric or duodenal ulcer was reported in 8.4% of patients who received sequential use of sorafenib.^20^ However, the incidence of diarrhea of grade 3/4 ranged from 6% to 8% and the grade 3/4 of anorexia and nausea was 0% to 2% in patients treated with sorefenib only.^7,8^ These data suggest the percentage of GI adverse effects were higher in multiple modalities.

The classic symptoms of infectious esophagitis include dysphagia, odynophagia, and retrosternal pain on swallowing.^4^ It can cause candida esophagitis when patients with impaired immunity, with chronic disease or under medications, such as gastric acid suppression therapy, malignancy, human immunodeficiency virus disease, illnesses characterized by immunodeficiency, DM, corticosteroid therapy, recurrent antibiotic use, prescribed chemotherapy and/or RT, proton pump inhibitors, H2-receptor antagonists, and prior vagotomy produce hypochlorhydria, which alters the colonization of the stomach by oral cavity bacteria and yeast and is thought to increase the risk of infectious esophagitis.^1–4,21,22^ The prevalence of esophageal candidiasis is 0.8% to 1.2%.^4,23^ In the current report, both patients under concurrent RT and sorafenib suffered from hiccups, epigastric distress, anorexia, heart burn, or retrosternal pain on swallowing and fatigue that were similar those of GI upset caused by sorafenib. Furthermore, we reviewed the records for 44 patients under such schedule in our institute retrospectively, 3/44 (6.8%, including 2 patients reported here) had epigastric distress or anorexia with panendoscopy-proved esophageal candidiasis.

Zhao et al^9^ found that sorafenib suppressed CD 4^+^ T-cell activation, proliferation, and cytokine production and induced T-cell cycle arrest and apoptosis in a dose-dependent manner. Hipp et al^24^ observed that sorafenib inhibited DCs antigen presentation, DC migration and their capability to stimulate primary T-cell responses by reducing the secretion of cytokines and the expression of major histocompatibility complex and CD1a molecules. These inhibitory effects were found to be mediated by the inhibition of phosphatidylinositol 3-kinase (PI3K), mitogen-activated protein kinase (MAPK), and nuclear factor kappa-light-chain-enhancer of activated B cells (NF-κB) signaling. These findings provide evidence that sorafenib suppresses the immune system and therefore increases the risk for infections due to candida species.

Opsonized candida species are ingested by both monocytes and monocyte-derived macrophages, but uptake of unopsonized candida is mediated only by monocyte-derived macrophages.^25,26^ Additionally, IFN-γ is one of the major factors that augment the phagocytic and candidacidal activities of human macrophages.^27^ Recently, Tsai et al^16^ reported that local irradiation, no matter daily dose or off-target dose, modulates the area under the concentration versus time curve of anticancer drugs in plasma. Furthermore, a growing body of evidence shows that irradiation has direct DNA damage-dependent effects, sending signals to distant normal tissues via a process known as the abscopal effect. The effect leads to overall genomic instability and radiation susceptibility in surrounding and distant normal tissues.^12,13^ Interestingly, fractionated irradiation has been shown in an animal model to suppress helper T1 (Th1) cytokine profiles, namely IFN-γ and the IFN-γ-inducible 10 kDa protein (IP-10).^14^ Song et al^15^ also found that the percentages of DCs and macrophages were also lower after fractionated irradiation in an animal model. After patients recovered from the episode and in the sequential maintaining course with sorafenib only, there was no recurrent esophageal candidiasis. Putting these published observations together, it is apparent that irradiation could modulate the concentration of anticancer drugs with abscopal effects that hint the effects of sorafenib may be modulated when concurrent with RT and it may cause the response of nonirradiation area similar with the irradiation area.

Sorafenib was shown to significantly enhance the sensitivity of the human HCC cell line SMMC-7721 to radiation in a schedule-dependent manner.^10,11^ Moreover, there is evidence that irradiation can induce the compensatory activation of multiple intracellular signaling pathway mediators, such as PI3K, MAPK, VEGF, c-jun N-terminal kinase (JNK), and NF-κB.^28^ The sorafenib-mediated blockade of the Raf/MAPK and VEGFR pathways therefore may enhance the efficacy of radiation.^29^ The evidence suggests that sorafenib with fractionated irradiation or sorafenib followed RT delivered sequentially provide better results but may enhance the adverse effects associated with each treatment modality, thereby decreasing the phagocytic and candidacidal activities of antifungal drugs due to radiosensitization.

Esophagogastroduodenoscopy with brushings or biopsy is currently the most sensitive and specific method for diagnosing candida esophagitis. The infection is characterized by the presence of patchy, whitish plaques covering a friable, erythematous mucosa.^23,30^ For immunosuppressed patients with candida esophagitis, the recommended drug is oral fluconazole with a loading dose of 400 mg followed by 200 to 400 mg once daily for 2 to 3 weeks without local antifungal therapy.^1,31^ In our patients, the epigastric and chest distress was improved after prescribed oral fluconazole accordingly.

## CONCLUSION

To the best of our knowledge this is the first report to show that treatment with sorafenib concurrent with RT or following RT can result in candida esophagitis. Physicians should be aware that sorafenib and RT can synergistically induce an immunosuppressive state in patients with HCC, thereby increasing their risk for esophagitis due to candida species.
